# Facile Synthesis of Oleanolic Acid Monoglycosides and Diglycosides

**DOI:** 10.3390/molecules13071472

**Published:** 2008-07-22

**Authors:** Yu Sha, Mao-Cai Yan, Jiao Liu, Yang Liu, Mao-Sheng Cheng

**Affiliations:** Key Lab of New Drug Design and Discovery of Liaoning Province, School of Pharmaceutical Engineering, Shenyang Pharmaceutical University, Shenyang 110016, P. R. China; E-mails: syna2000@yahoo.com (Y. S.); yanmaocai@126.com (M.-C. Y.); amityliu@163.com (Y. L.); ly_99@sina.com (Y. L.)

**Keywords:** Oleanolic acid, triterpenoid, glycoside, synthesis, glycosylation

## Abstract

Oleanolic acid and its glycosides are important natural products, possessing various attractive biological activities such as antitumor, antivirus and anti-inflammatory properties. In the present work, fifteen oleanolic acid saponins bearing various saccharide moieties, including 3-monoglycoside, 28-monoglycoside and 3,28-diglycoside, were easily synthesized in high yields. Benzyl was chosen as the protective group for the COOH(28) group, instead of commonly used methyl and allyl, to avoid difficulties in the final deprotection. Alkali-promoted condensation of the carboxylic acid with bromo-glycosides was found to be more efficient in the synthesis of 28-glycosides. Two approaches were investigated and proved practicable in the preparation of 3,28-diglycosides. This method is suitable for preparing oleanolic acid glycosides with structural diversity for extensive biological evaluation and structure-activity relationship study, and it also apply new idea for the corresponding synthetic methods to the glycoside derivatives of other triterpenoid.

## Introduction

**Figure 1:**
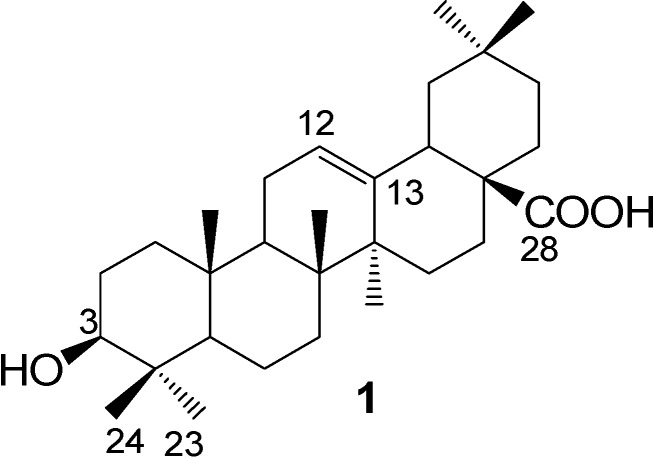
Structure of oleanolic acid

Oleanolic acid (OA, **1**, Figure 1) is one of the most important triterpenes, which is found widely distributed in Nature, along with its glycosides [1]. Possessing multiple biological effects, such as antitumor, antiulcer, antivirus, antihyperlipidemic, anti-inflammatory and hepatoprotective activities [1,2,3,4,5], OA and its saponins have received increasing attentions in recent years. The saccharide moiety of OA saponins has been revealed to be of importance for their bioactivities. For example, oleanane-type triterpenoid saponins bearing an *α*-L-rhamnopyranosyl-(1→2)-*α*-L-arabinopyranosyl moiety at C(3)-OH generally show markedly higher antitumor activity than their aglycons [4,5]. Some OA glycosides show significant gastrointestinal transit acceleration or inhibition activity in mice, while the aglycon OA does not [6].

Although OA has become an auxiliary drug in the treatment of liver disease, many bioactivities of OA glycosides are too weak to be utilized in clinical therapy. Chemical synthesis and modification is known to be a powerful tool in preparation of novel compounds with diversity for pharmacology studies and new chemical entity development. In fact, the synthesis of OA glycosides has attracted much attention from researchers for a long time. As early as 1952, Hardegger *et al.* reported the glycosylation of OA esters with acetylated bromoglycosides [7]. Later a few studies were reported on the glycosylation of OA by the Koenigs-Knorr method [8,9,10,11,12,13], which was usually inefficient and resulted in uneven yields. Although methyl was most commonly used as protective group for the carboxylic acid in the early synthesis work, alkaline hydrolysis of OA esters was very difficult due to the high steric hinderance [14]. Therefore, halolysis (usually LiI/DMF, reflux) was often employed to cleave OA esters, but was of low efficiency and too harsh for many groups. In 1999, Deng *et al.* reported a highly efficient glycosylation of triterpenoids and steroids *via* the Schmidt method [15,16], in which the allyl ester of OA was glycosylated with benzoylated trichloroacetimidates in very high yields (>90%). Based on this method, Zang *et al.* accomplished in 2005 the synthesis of four 3-mono-glycosides of OA [17]. The glycosylation of allyl oleanolate was carried out in 80~90% yield, however, removal of allyl in the final step by treatment with PdCl_2_ for 24 h afforded relatively lower yields (only 41~47%). A few syntheses of some bioactive OA saponins bearing complex saccharide moieties were accomplished in recent years [18,19,20], nevertheless more facile and efficient synthetic methods towards this interesting structure require further investigation. Based on our experience in triterpenoid saponin synthesis, we started with the highly efficient preparation of OA glycosides on a large scale for extensive bioactivity evaluation and structure-activity relationship study.

## Results and Discussion

OA has two glycosylation sites, *i.e.* C(3)-OH and COOH(28), which can be glycosylated to form 3-monoglycosides, 28-monoglycosides and 3,28-diglycosides. The present work describes the initial glycosylation study of OA, which was carried out with some commercially available sugars, such as D-glucose, L-arabinose, lactose and maltose.

### 1. Preparation of OA 3-glycosides

Since common alkyl esters of OA are difficult to retransform into free carboxylic acids, selection of a suitable protective group for COOH(28) is the primary problem. Trityl (Tr) and tert-butyl-diphenylsilyl (TBDPS) had been used in the previous work [21,22], however, they are too sensitive to act as permanent protective groups in the preparation of complex glycosides. As mentioned above, the deprotection was usually inefficient when allyl was employed [17,18]. In our previous work on triterpenoid saponin synthesis [19,23], benzyl was chosen and proved to be an excellent permanent protective group for COOH(28), as it can be conveniently removed through catalytic hydrogenolysis in nearly quantitive yields, while the double bond between C(12) and C(13) would not be affected [24].Therefore, we adopted benzyl in the present work.

**Scheme 1:**
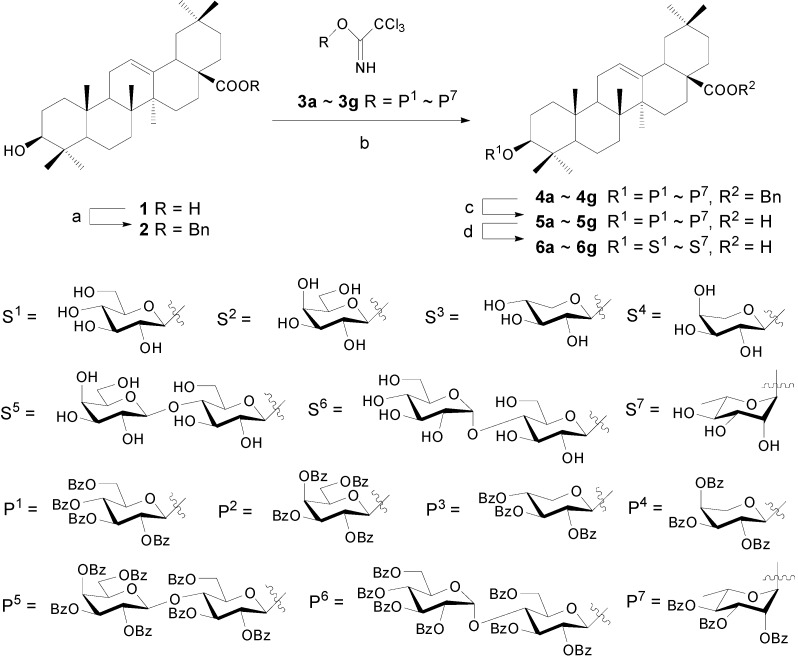
Synthesis of OA 3-glycosides.

*Reagents and conditions*: (a) BnBr, K_2_CO_3_, THF-H_2_O, rt; (b) TMSOTf, 4 Å MS, CH_2_Cl_2_, rt; (c) H_2_, Pd-C (10%), EtOAc, reflux; (d) NaOMe, CH_2_Cl_2_-MeOH, rt.

As shown in Scheme 1, OA was first converted into its benzyl ester **2** in 98% yield [23], and C(3)-OH was then glycosylated with trichloroacetimidates **3** under promotion with TMSOTf. The benzyl moiety was then removed under catalytic hydrogenolysis to give free carboxylic acids **5**, which would be subjected to further glycosylation to give 3,28-diglycosides in the later work. Removal of benzoyl on **5** through ester exchange in NaOMe-MeOH afforded target OA 3-glycosides **6**. According to this method, seven 3-glycosides of OA were easily prepared in overall yields of 58%~79%.

### 2. Preparation of OA 28-glycosides

**Scheme 2:**
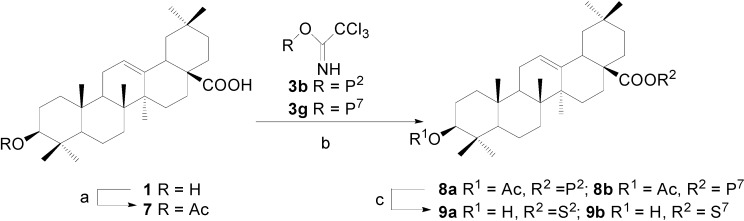
Synthesis of OA 28-glycosides (the first approach).

Two approaches were attempted to prepare OA 28-glycosides. In the first approach, the C(3)-OH of OA was acetylated in Ac_2_O-pyridine and glycosylation of COOH(28) was carried out with benzoylated trichloroacetimidates. Benzoyl and acetyl groups were then removed by NaOMe-MeOH to give OA 28-glycosides (Scheme 2). However, removal of Ac on C(3)-OH was found to be more difficult than that of benzoyl groups on the saccharide moieties due to the steric hinderance from C(23) and C(24). Stronger alkaline condition usually led to the partial breakage of acyl glycoside linkage at C(28). By this approach OA ester of *β*-D-galactose (**9a**) and *α*-L-rhamnose (**9b**) were synthesized in 18% and 21% total yields, respectively.

*Reagents and conditions*: (a) Ac_2_O, pyridine, rt; (b) TMSOTf, 4 Å MS, CH_2_Cl_2_, 0°C; (c) NaOMe, CH_2_Cl_2_-MeOH, rt.

**Scheme 3:**
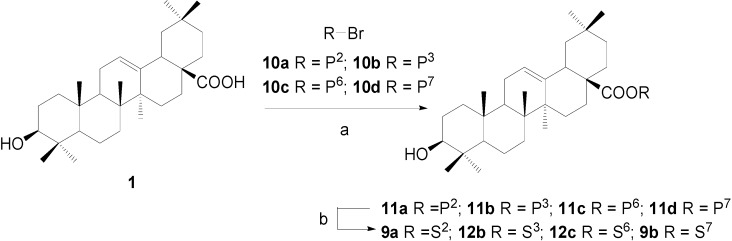
Synthesis of OA 28-glycosides (the second approach).

*Reagents and conditions*: (a) K_2_CO_3_, CHCl_3_-H_2_O, Bu_4_NBr, 50°C; (b) NaOMe, CH_2_Cl_2_-MeOH, rt.

Alternatively, alkaline-promoted condensation of free OA with benzoylated bromoglycosides (R-Br, R=P^2^, P^3^, P^6^, and P^7^) under phase transfer catalysis was attempted (Scheme 3) [25]. However, no significant reaction was observed under the reported conditions, possibly because benzoylated bromoglycosides are more stable than the acetylated donors employed in the literature. When modified conditions (K_2_CO_3_, Bu_4_NBr, CH_2_Cl_2_-H_2_O, reflux) were used [22], the reaction proceeded very slowly and the decomposed bromoglycoside was the main by-product mixed with the desired compound. As improvements, the solvent dichloromethane was replaced by chloroform and the turbid system was heated up to 50°C and stirred vigorously to successfully give the glycosylation products. All the benzoyl groups on the sugar parts were removed by NaOMe/MeOH, while the acyl glycoside linkages were not affected under these conditions. By this approach, glycosides **9a** and **9b** were synthesized once more in higher yields of 80% and 78%. Furthermore, other two 28-glycosides (**12b**, **12c**) were readily prepared from OA in 74% and 70% yields, respectively. Understandably, due to the relatively less efficiency of Schmidt glycosylation of carboxylic acid and low selectivity in the deprotection in the first approach, the second approach was more preferable for the preparation of OA 28-glycosides.

### 3. Preparation of OA 3,28-diglycosides

Many natural occurring saponins of OA with important biological activities are 3,28-diglycosides. Two approaches were also investigated in the preparation of this type of structures. The first approach was to introduce one saccharide moiety at C(3)-OH after protection of COOH(28) with benzyl, and to attach another saccharide moiety to the carboxylic acid after removal of the benzyl. The second approach was to introduce one saccharide to COOH(28) using a bromoglycoside under alkaline conditions, then C(3)-OH was to be glycosylated with a trichloroacetimidate. In the present work, intermediates **5** and **11** in the preparation of 3-glycosides and 28-glycosides were utilized in the synthesis of OA 3,28-diglycosides through these two approaches, respectively.

**Scheme 4:**
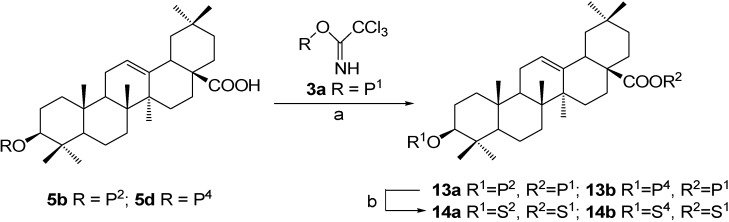
Synthesis of OA 3,28-diglycosides (the first approach).

*Reagents and conditions*: (a) TMSOTf, 4 Å MS, CH_2_Cl_2_, 0°C~rt; (b) NaOMe, CH_2_Cl_2_-MeOH, rt.

As shown in Scheme 4, 3-*O*-(2,3,4,6-tetra-*O*-benzoyl-*β*-D-galactopyranosyl)oleanolic acid (**5b**) was subjected to Schmidt glycosylation with trichloroacetimidate **3a** to give the full protected 3,28-diglycoside **13a**, which was then treated with NaOMe-MeOH to afford target product *β*-D-glucopyranosyl oleanolate 3-*O*-*β*-D-galactopyranoside (**14a**). According to the same procedure, *β*-D-glucopyranosyl oleanolate 3-*O*-*α*-L-arabinopyranoside (**14b**) was prepared from intermediate **5d**.

**Scheme 5:**
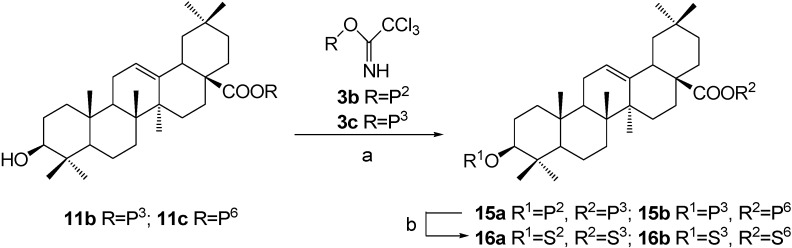
Synthesis of OA 3,28-diglycosides (the second approach).

In the second approach, the intermediate 2,3,4-tri-*O*-benzoyl-*β*-D-xylopyranosyl oleanolate (**11b**) was subjected to Schmidt glycosylation with **3b** to introduce a galactose residue to the free C(3)-OH (Scheme 5). Removal of benzoyl groups afforded target *β*-D-xylopyranosyl oleanolate 3-*O*-*β*-D-galactopyranoside (**16a**). According to the same procedure, *α*-D-glucopyranosyl-(1→4)-*β*-D-glucopyranosyl oleanolate 3-*O*-*β*-D-xylopyranoside (**16b**) was synthesized from intermediate **11c** and trichloroacetimidate **3c**.

*Reagents and conditions*: (a) TMSOTf, 4 Å MS, CH_2_Cl_2_, rt; (b) NaOMe, CH_2_Cl_2_-MeOH, rt.

It is obvious that the second approach to 3,28-diglycosides is preferable to the first one, not only due to its conciseness in avoiding introduction and removal of benzyl group, but also because alkaline-promoted condensation was more efficient in the glycosylation of carboxylic acid. However, the first approach also affords good yields and it is applicable in certain cases. For instance, in the synthesis of glycoside analogues with the same 3-*O*-saccharide moiety, or in preparation of glycosides in large amounts, it can make use of various intermediates with free carboxylic acids.

The 15 glycosides prepared in the present work and the overall yields from OA are listed in Table 1. All glycosidic linkages formed in the glycosylation are 1,2-*trans*-, due to the neighboring participating effect of benzoyl at C(2)-OH of saccharide donors, which can also be confirmed by the *J* values of hydrogen on the anomeric carbon of the saccharide moiety. In ^1^H-NMR spectra of the synthetic saponins, the 1,2-trans- configuration of the anomeric carbons of all glucosyl, gallactosyl, arabinosyl and xylosyl units were determined by the *J*_H1-H2_ values (above 6.0 Hz), while the *J*_H1-H2_ values (1.0-1.5 Hz) of all rhamnosyl units indicated the alpha-configuration of their anomeric carbons. For compound 6a, its 7.7 Hz *J* value of the hydrogen on anomeric carbon with characterized 4.93 ppm chemical shift indicates that two hydrogens on C(1') and C(2') of glucose are 1,2-*trans*-configuration. And in the case of 9b, its 1.4 Hz *J* value of the hydrogen on anomeric carbon (5.43 ppm) also indicates that two hydrogens on C(1') and C(2') of rhamnose are 1,2-*trans*-configuration.

**Table 1:** OA glycosides and overall yields from OA.

Compound	C(3) saccharide	C(28) saccharide	Total yield from OA	Literature yield
**6a**	β-D-Glc	H	58%	25% [17]
				36% [11]
**6b**	β-D-Gal	H	55%	24% [17]
**6c**	β-D-Xyl	H	71%	26% [17]
**6d**	α-L-Ara	H	76%	25% [17]
**6e**	β-D-Gal-(1→4)-β-D-Glc	H	75%	4.0% [13]
**6f**	α-D-Glc-(1→4)-β-D-Glc	H	69%	–
**6g**	α-L-Rha	H	79%	–
**9a**	H	β-D-Gal	18% (Scheme 2)	not presented
			80% ( Scheme 3)	[25]
**12b**	H	β-D-Xyl	74%	54% [10]
**12c**	H	α-D-Glc-(1→4)-β-D-Glc	70%	–
**9b**	H	α-L-Rha	21% (Scheme 2)	–
			78% (Scheme 3)	
**14a**	β-D-Gal	β-D-Glc	28%	–
**14b**	α-L-Ara	β-D-Glc	54%	–
**16a**	β-D-Gal	β-D-Xyl	66%	–
**16b**	β-D-Xyl	α-D-Glc-(1→4)-β-D-Glc	44%	–

## Conclusions

In conclusion, 15 OA glycosides, including 3-monoglycosides, 28-monoglycosides and 3,28-diglycosides, were easily synthesized in high yields. This method is suitable for preparing OA glycosides on a large scale and analogues for extensive biological evaluation, mechanism research and structure-activity relationship study. Moreover, this work may also provide an efficient method in preparing glycoside derivatives of other pentacyclic triterpenoid carboxylic acids, such as ursolic acid, glyrrhetinic acid and boswellic acid. Related work in this field is currently in progress and will be reported in due course.

## Experimental

### General

Commercial reagents were used without further treatment unless specialized. Solvents were dried and distilled prior to use in the usual way. Boiling range of petroleum ether was 60~90°C. Analytical TLC was performed with silica gel GF_254_. Preparation column chromatography was performed with silica gel H. Saccharide donors (trichloroacetimidates and bromoglycosides) were prepared according to the reported methods [16,25,26]. ^1^H-NMR and ^13^C-NMR spectra were recorded on a Bruker ARX 300 MHz instrument. *J* values were given in Hz. ESI-MS were obtained on an Agilent 1100 mass spectrometer. HRMS was detected on High resolution ESI-FTICR mass spectrometry (Ion spec 7.0T).

### Synthesis of OA 3-glycosides **6a**~**6g**

### Benzyl oleanolate (**2**).

A suspension of OA (5.00 g, 10.9 mmol), BnBr (2.10 mL, 17.5 mmol) and K_2_CO_3_ (3.00 g, 21.8 mmol) in THF-H_2_O (40:1, 82 mL) was stirred overnight at rt. The mixture was then filtered, and the filtrate was concentrated under vacuum and purified through a silica gel column chromatography (8:1, petroleum ether-EtOAc) to give **2** (5.84 g, 98%) as a white amorphous solid. The physical data agreed with that previously reported [19].

### Oleanolic acid 3-O-α-L-arabinopyranoside (**6d**)

Benzyl ester **2** (200 mg, 0.366 mmol), trichloroacetimidate **3d** (244 mg, 0.402 mmol) and powdered 4 Å molecular sieves (500 mg) were stirred for 30 min at rt in dry CH_2_Cl_2_ (6 mL). A solution of TMSOTf in dry CH_2_Cl_2_ (1%, 0.35 mL) was added dropwise. The mixture was stirred for 20 min followed by addition of Et_3_N (0.10 mL) and filtration. The filtrate was concentrated and purified by a silica gel column chromatography (8:1, petroleum ether-EtOAc) to afford benzyl oleanolate 3-*O*-(2,3,4-tri-*O*-benzoyl-*α*-L-arabinopyranoside) (**4d**) (330 mg, 91%) as a white amorphous solid. *R*_f_ = 0.65 (4:1, petroleum ether-EtOAc); ^1^H-NMR (CDCl_3_) δ 8.09-7.26 (*m*, 20H, H-C(Ar)), 5.75 (*dd*, *J=*8.8, 6.5, 1H, H-C(2')), 5.67 (*m*, 1H, H-C(4')), 5.59 (*dd*, *J=*8.9, 3.5, 1H, H-C(3')), 5.30 (*t*, *J=*3.0, 1H, H-C(12)), 5.06 (*dd*, *J=*19.9, 12.6, 2H, PhC*H*_2_), 4.79 (*d*, *J=*6.6, 1H, H-C(1')), 4.33 (*dd*, *J=*12.9, 3.8, 1H, H-C(5')-1), 3.87 (*m*, 1H, H-C(5')-2), 3.16 (*dd*, *J=*11.0, 4.9, 1H, H-C(3)), 2.90 (*dd*, *J=*10.1, 3.2, 1H, H-C(18)), 1.11, 0.92, 0.90, 0.86, 0.79, 0.66, 0.58 (*s*, 7×3H, CH_3_); ESI-MS: 1013.5 [(M+Na)^+^].

A suspension of **4d** (200 mg, 0.202 mmol) and 10% Pd-C (32 mg) in EtOAc (8 mL) was refluxed and bubbled up with H_2_ (20 mL/min) for 3 h. The mixture was then filtered and the filtrate was concentrated to dryness to afford oleanolic acid 3-*O*-(2,3,4-tri-*O*-benzoyl-*α*-L-arabinopyranoside) (**5d**) (180 mg, 99%) as a white amorphous solid. *R*_f_ = 0.36 (3:1, petroleum ether-EtOAc); ^1^H-NMR (CDCl_3_) δ 8.07-7.18 (*m*, 15H, H-C(Ar)), 5.77 (*dd*, 1H, *J=*8.8, 6.4, H-C(2')), 5.67 (*m*, 1H, H-C(4')), 5.59 (*dd*, 1H, *J=*8.8, 3.5, H-C(3')), 5.26 (*br s*, 1H, H-C(12)), 4.78 (*d*, 1H, *J=*6.3, H-C(1')), 4.33 (*dd*, 1H, *J=*13.0, 3.9, H-C(5')-1), 3.87 (*dd*, 1H, *J=*13.0, 1.9, H-C(5')-2), 3.16 (*dd*, 1H, *J=*11.1, 4.8, H-C(3)), 2.80 (*dd*, 1H, *J=*9.9, 2.6, H-C(18)), 1.10, 0.92, 0.90, 0.87, 0.77, 0.70, 0.62 (*s*, 7×3H, CH_3_); ESI-MS: 918.8 [(M+NH_4_)^+^].

Compound **5d** (84 mg, 0.093 mmol) was suspended in dry MeOH (5 mL), to which a freshly prepared solution of NaOMe in MeOH (1.0 M, 0.40 mL) was added. The mixture was stirred at rt overnight and then neutralized with Dowex H^+^ resin to pH 7 and filtered. The filtrate was concentrated and purified with a silica gel column chromatography (9:1, CHCl_3_-MeOH) to give oleanolic acid 3-*O*-*α*-L-arabinopyranoside (**6d**) (47 mg, 85%) as a white powder. *R*_f_ = 0.56 (5:1, CHCl_3_-MeOH); ^1^H-NMR (pyridine-*d_5_*) δ 5.47 (*br s*, 1H, H-C(12)), 4.77 (*d*, 1H, *J=*7.0, H-C(1')), 4.43 (*t*, 1H, *J=*8.7), 4.31 (*m*, 2H), 4.16 (*dd*, 1H, *J=*8.7, 3.1), 3.83 (*m*, 1H), 3.37-3.27 (*m*, 2H, H-C(3), H-C(18)), 1.29, 1.27, 1.00, 0.99, 0.94, 0.93, 0.83 (*s*, 7×3H, CH_3_); ^13^C-NMR (pyridine-*d_5_*) δ 180.2, 144.8, 122.6, 107.6, 88.7, 74.7, 73.0, 69.6, 66.9, 55.9, 48.1, 46.7, 46.5, 42.2, 42.0, 39.8, 39.6, 38.8, 37.0, 34.2, 33.3, 33.2, 33.2, 31.0, 28.2, 28.2, 26.7, 26.2, 23.8, 23.8, 23.7, 18.5, 17.4, 16.9, 15.5; ESI-MS: 611.5 [(M+Na)^+^], 627.3 [(M+K)^+^]. HRMS: *m*/*z* 587.3962 [M-H]^-^ ([C_35_H_55_O_7_] = 587.3953).

Compounds **6a~6c** and **6e~6g** were prepared according to the same procedure described for **6d**.

*Oleanolic acid 3-O-β-D-glucopyranoside* (**6a**). *R*_f_ = 0.39 (5:1, CHCl_3_-MeOH); ^1^H-NMR (pyridine-*d_5_*) δ 5.44 (*br s*, 1H, H-C(12)), 4.93 (*d*, 1H, *J=*7.7, H-C(1')), 4.57 (*br d*, 1H, *J=*11.3), 4.43 (*m*, 1H), 4.25 (*m*, 2H), 4.01 (*m*, 2H), 3.46 (*dd*, 1H, *J=*12.5, 2.2, H-C(18)), 3.37 (*dd*, 1H, *J=*8.0, 2.4, H-C(3)), 1.31, 1.30, 1.01, 0.98, 0.93, 0.91, 0.80 (*s*, 7×3H, CH_3_); ^13^C-NMR (pyridine-*d_5_*) δ 180.2, 145.4, 122.4, 107.0, 88.8, 78.8, 78.5, 78.4, 71.8, 63.0, 55.8, 48.0, 46.8, 46.7, 42.2, 42.1, 39.7, 39.5, 38.7, 37.0, 34.4, 33.4, 33.3, 33.0, 31.1, 30.0, 29.9, 28.3, 26.2, 23.9, 23.9, 23.8, 18.5, 17.5, 17.1, 15.5; ESI-MS: 641.4 [(M+Na)^+^], 653.4 [(M+Cl)^-^]. HRMS: *m*/*z* 617.4071 [M-H]^-^ ([C_36_H_57_O_8_] = 617.4059).

*Oleanolic acid 3-O-β-D-galactopyranoside* (**6b**). *R*_f_ = 0.40 (5:1, CHCl_3_-MeOH); ^1^H-NMR (DMSO-*d_6_*) δ12.07 (*s*, 1H, COOH), 5.16 (*br s*, 1H, H-C(12)), 4.75, 4.64, 4.52, 4.33 (*br s*, 4×1H, OH), 4.11 (*d*, 1H, *J=*6.4, H-C(1')), 3.62 (*br s*, 1H), 3.52 (*m*, 1H), 3.43 (*m*, 1H), 3.29-3.25 (*m*, 3H), 3.03 (*br d*, *J=*7.8, H-C(3)), 2.74 (*br d*, 1H, *J=*10.6, H-C(18)), 1.10 (*s*, 3H, CH_3_), 0.98 (*s*, 3H, CH_3_), 0.88 (*s*, 9H, 3×CH_3_), 0.76 (*s*, 3H, CH_3_), 0.72 (*s*, 3H, CH_3_); ^13^C-NMR (DMSO-*d_6_*) δ 178.7, 143.9, 121.6, 106.2, 88.0, 75.0, 73.7, 71.2, 68.2, 60.4, 55.1, 47.2, 45.8, 45.6, 41.4, 40.9, 38.7, 38.3, 38.2, 36.4, 33.4, 32.9, 32.5, 32.2, 30.5, 27.8, 27.3, 25.7, 25.6, 23.5, 23.0, 22.7, 17.9, 16.9, 16.6, 15.2; ESI-MS: 641.4 [(M+Na)^+^]. HRMS: *m*/*z* 617.4062 [M-H]^-^ ([C_36_H_57_O_8_] = 617.4059).

*Oleanolic acid 3-O-β-D-xylopyranoside* (**6c**). *R*_f_ = 0.69 (5:1, CHCl_3_-MeOH); ^1^H-NMR (pyridine-*d_5_*) δ 5.45 (*br s*, 1H, H-C(12)), 4.84 (*d*, 1H, *J=*7.5, H-C(1')), 4.39 (*dd*, 1H, *J=*11.2, 4.9), 4.25-4.15 (*m*, 2H), 4.03 (*t*, 1H, *J=*7.8), 3.79 (*t*, 1H, *J=*10.4), 3.44 (*br d*, 1H, *J=*10.3, H-C(18)), 3.35 (*dd*, 1H, *J=*11.2, 3.8, H-C(3)), 1.31, 1.30, 1.02, 0.98, 0.95, 0.94, 0.85 (*s*, 7×3H, CH_3_); ^13^C-NMR (pyridine-*d_5_*) δ 180.4, 144.9, 122.5, 107.7, 88.6, 78.7, 75.6, 71.3, 67.2, 55.9, 48.1, 46.7, 46.5, 42.2, 42.0, 39.7, 39.6, 38.8, 37.0, 34.3, 33.3, 33.3, 33.2, 31.0, 28.3, 28.2, 26.8, 26.2, 23.8, 23.8, 23.7, 18.5, 17.4, 17.0, 15.5; ESI-MS: 611.5 [(M+Na)^+^]. HRMS: *m*/*z* 587.3953 [M-H]^-^ ([C_35_H_55_O_7_] = 587.3953).

*Oleanolic acid 3-O-β-D-galactopyranosyl-(1→4)-β-D-glucopyranoside* (**6e**). *R*_f_ = 0.16 (4:1, CHCl_3_-MeOH); ^1^H-NMR (DMSO-*d_6_*) 5.16 (*br s*, 1H, H-C(12)), 4.23-4.21 (*m*, 2H, H-C(1'), H-C(1'')), 3.73-3.69 (*br d*, 1H, *J=*11.0), 3.61-3.44 (*m*, 5H), 3.33-3.25 (*m*, 5H), 3.04-3.01 (*m*, 2H, H-C(2'), H-C(3)), 2.73 (*dd*, 1H, *J=*12.8, 3.4, H-C(18)), 1.09 (*s*, 3H, CH_3_), 0.98 (*s*, 3H, CH_3_), 0.87 (*s*, 9H, 3×CH_3_), 0.75 (*s*, 3H, CH_3_), 0.71 (*s*, 3H, CH_3_); ^13^C-NMR (DMSO-*d_6_*) δ 178.8, 144.0, 121.7, 105.3, 104.1, 88.3, 81.3, 75.6, 75.2, 74.7, 73.7, 73.3, 70.7, 68.2, 60.8, 60.5, 55.2, 47.3, 45.9, 45.6, 41.5, 40.9, 38.8, 38.7, 38.3, 36.5, 33.5, 33.0, 32.6, 32.3, 30.6, 27.8, 27.4, 25.8, 25.8, 23.5, 23.1, 22.8, 18.0, 17.0, 16.7, 15.3; ESI-MS: 781.0 [(M+H)^+^], 798.0 [(M+NH_4_)^+^]. HRMS: *m*/*z* 779.4580 [M-H]^-^ ([C_42_H_67_O_13_] = 779.4587).

*Oleanolic acid 3-O-α-D-glucopyranosyl-(1→4)-β-D-glucopyranoside* (**6f**). *R*_f_ = 0.13 (4:1, CHCl_3_-MeOH); ^1^H-NMR (pyridine-*d_5_*) δ 5.78 (*d*, 1H, *J=*3.5, H-C(1'')), 5.50 (*br s*, 1H, H-C(12)), 5.32 (*br d*, 1H, *J=*11.5), 5.08 (*dd*, 1H, *J=*9.5, 5.6), 4.82 (*d*, 1H, *J=*7.7, H-C(1')), 4.63-4.50 (*m*, 3H), 4.43-4.31 (*m*, 2H), 4.24-4.04 (*m*, 5H), 3.28 (*m*, 2H, H-C(3), H-C(18)), 1.29, 1.25, 1.01, 0.96, 0.95, 0.90, 0.76 (*s*, 7×3H, CH_3_); ^13^C-NMR (pyridine-*d_5_*) δ 180.2, 144.9, 122.6, 106.8, 103.8, 89.6, 82.9, 77.9, 75.6, 75.4, 74.9, 74.4, 73.3, 71.6, 65.1, 62.5, 55.9, 48.0, 46.7, 46.5, 42.2, 42.0, 39.7, 39.4, 38.5, 36.9, 34.3, 33.3, 33.2, 33.2, 31.0, 28.3, 28.2, 26.5, 26.3, 23.8, 23.8, 23.7, 18.5, 17.4, 16.9, 15.4; ESI-MS: 779.2 [(M-H)^-^], 803.4 [(M+Na)^+^]. HRMS: *m*/*z* 779.4584 [M-H]^-^ ([C_42_H_67_O_13_] = 779.4587).

*Oleanolic acid 3-O-α-L-rhamnopyranoside* (**6g**). *R*_f_ = 0.80 (5:1, CHCl_3_-MeOH); ^1^H-NMR (DMSO-*d_6_*) δ 12.05 (*s*, 1H, COOH), 5.16 (*br s*, 1H, H-C(12)), 4.70 (*m*, 2H, H-C(1'), OH), 4.58 (*s*, 1H, OH), 4.52 (*d*, 1H, *J=*5.8, OH), 3.62 (*br s*, 1H), 3.49 (*m*, 1H), 3.40 (*m*, 1H), 3.17 (*m*, 1H), 3.01 (*dd*, 1H, *J=*9.7, 3.0, H-C(3)), 2.74 (*br d*, 1H, *J=*10.8, H-C(18)), 1.10 (*s*, 3H, CH_3_), 1.09 (*s*, 3H, CH_3_), 0.87 (*s*, 9H, 3×CH_3_), 0.72 (*s*, 6H, 2×CH_3_); ^13^C-NMR (DMSO-*d_6_*) δ 178.7, 143.9, 121.6, 103.0, 87.6, 72.2, 70.8, 70.8, 68.6, 54.7, 47.1, 45.8, 45.5, 41.4, 40.9, 38.8, 38.6, 37.9, 36.4, 33.4, 32.9, 32.4, 32.2, 30.5, 28.0, 27.3, 25.7, 25.0, 23.5, 23.0, 22.7, 17.9, 17.8, 16.9, 16.5, 15.2; ESI-MS: 637.6 [(M+Cl)^-^]. HRMS: *m*/*z* 601.4104 [M-H]^-^ ([C_36_H_57_O_7_] = 601.4110).

### Synthesis of OA 28-glycosides **9a~9b**, approach 1: β-D-galactopyranosyl oleanolate (**9a**)

Ac_2_O (18.9 mL) was added dropwise to a solution of OA (9.14 g, 20 mmol) in dry pyridine (38 mL) at 0°C under stirring. To the mixture was added DMAP (244 mg, 2 mmol) and the mixture was allowed to warm up to rt and stirred overnight. Water (15 mL) was added to quench the reaction. The mixture was then concentrated in vacuum and the residue was dissolved in CH_2_Cl_2_ (150 mL) and washed with 5% HCl, saturated NaHCO_3_ and brine in sequence. The solution was dried over Na_2_SO_4_. Recrystallization with MeOH-CH_2_Cl_2_ gave oleanolic acid 3-acetate (**7**) (7.66 g, 77%) as a white powder. *R*_f_ = 0.78 (3:1, petroleum ether-EtOAc); ^1^H-NMR (CDCl_3_) δ 5.27 (*br s*, 1H, H-C(12)), 4.49 (*dd*, 1H, *J=*8.2, 7.5, H-C(3)), 2.82 (*br d*, 1H, *J=*10.0, H-C(18)), 2.05 (*s*, 3H, Ac), 1.12, 0.94, 0.93, 0.90, 0.86, 0.85, 0.74 (*s*, 7×3H, CH_3_); ESI-MS: 521.8 [(M+Na)^+^].

A suspension of **7** (250 mg, 0.501 mmol), **3b** (480 mg, 0.648 mmol) and powdered 4 Å molecular sieves (800 mg) in dry CH_2_Cl_2_ (8 mL) were stirred for 30 min at 0°C. TMSOTf (40 μL) was added and the mixture was stirred for 20 min before Et_3_N (0.15 mL) was added to quench the reaction. The mixture was then filtered and the filtrate was concentrated and purified by a silica gel column chromatography (7:1, petroleum ether-EtOAc) to afford 3-*O*-acetyloleanolic acid 2,3,4,6-tetra-*O*-benzoyl-*β*-D-galactopyranosyl ester (**8a**) (391 mg, 72%) as a white amorphous solid. *R*_f_ = 0.70 (4:1, petroleum ether-EtOAc); ^1^H-NMR (CDCl_3_) δ 8.10-7.21 (*m*, 20H, H-C(Ar)), 6.02 (*d*, 1H, *J=*3.3), 5.93 (*m*, 2H), 5.73 (*m*, 1H), 5.31 (*br s*, 1H, H-C(12)), 4.62 (*dd*, 1H, *J=*10.6, 6.1), 4.46-4.33 (*m*, 3H), 2.82 (*dd*, 1H, *J=*11.8, 2.1, H-C(18)), 2.04 (*s*, 3H, Ac), 0.97, 0.88, 0.85, 0.83, 0.82, 0.81, 0.49 (*s*, 7×3H, CH_3_); ESI-MS: 1099.8 [(M+Na)^+^].

**8a** (94 mg, 0.087 mmol) was dissolved in dry MeOH (8 ml), to which a freshly prepared solution of NaOMe in MeOH (1.0 M, 1.60 mL) was added. The mixture was stirred at rt for 12 h and then neutralized with Dowex H^+^ resin to pH 7 and filtered. The filtrate was concentrated and purified with a silica gel column chromatography (10:1, CHCl_3_-MeOH) to give *β*-D-galactopyranosyl oleanolate (**9a**) (17 mg, 32%) as a white powder. *R*_f_ = 0.43 (8:1, CHCl_3_-MeOH); ^1^H-NMR (pyridine-*d_5_*) δ 6.27 (*d*, 1H, *J=*8.0, H-C(1')), 5.44 (*br s*, 1H, H-C(12)), 4.69-4.64 (*m*, 2H), 4.48 (*m*, 1H), 4.40 (*m*, 1H), 4.21 (*m*, 2H), 3.43 (*dd*, 1H, *J=*9.5, 5.6, H-C(3)), 3.19 (*br d*, 1H, *J=*11.1, H-C(18)), 1.21, 1.21, 1.14, 1.02, 0.91, 0.88, 0.86 (*s*, 7×3H, CH_3_); ^13^C-NMR (pyridine-*d_5_*) δ 176.0, 143.7, 122.4, 95.8, 77.6, 77.3, 75.2, 71.0, 69.6, 61.4, 55.3, 47.7, 46.5, 45.7, 41.6, 41.3, 39.4, 38.9, 38.5, 36.9, 33.5, 32.7, 32.7, 32.0, 30.3, 28.3, 27.8, 27.6, 25.6, 23.4, 23.2, 22.8, 18.3, 17.0, 16.1, 15.2; ESI-MS: 641.4 [(M+Na)^+^]. HRMS: *m*/*z* 617.4058 [M-H]^-^ ([C_36_H_57_O_8_] = 617.4059).

### α-L-Rhamnopyranosyl oleanolate (**9b**)

This compound was prepared according to the same procedure described for **9a**. *R*_f_ =0.60 (8:1, CHCl_3_-MeOH); ^1^H-NMR (pyridine-*d_5_*) δ 6.79 (*s*, 1H, *J=*1.4, H-C(1')), 5.43 (*br s*, 1H, H-C(12)), 4.58 (*m*, 1H), 4.51 (*dd*, 1H, *J=*8.6, 3.1), 4.41-4.36 (*m*, 2H), 3.44 (*dd*, 1H, *J=*9.4, 6.1, H-C(3)), 3.14 (*br d*, 1H, *J=*10.2, H-C(18)), 1.70 (*d*, 3H, *J=*5.4, H-C(6')), 1.23, 1.20, 1.04, 1.02, 0.90, 0.90, 0.86 (*s*, 7×3H, CH_3_); ^13^C-NMR (pyridine-*d_5_*) δ 175.9, 143.9, 122.6, 95.4, 78.1, 73.4, 72.9, 72.6, 71.6, 55.8, 48.0, 47.4, 46.0, 42.2, 42.1, 39.8, 39.4, 39.0, 37.4, 33.9, 33.3, 33.1, 33.0, 30.9, 28.8, 28.1, 28.0, 26.0, 23.9, 23.6, 23.3, 18.8, 18.8, 17.7, 16.6, 15.6; ESI-MS: 637.6 [(M+Cl)^-^]. HRMS: *m*/*z* 601.4105 [M-H]^-^ ([C_36_H_57_O_7_] = 601.4110).

### Synthesis of OA 28-glycosides **12a~12d**, approach 2: β-D-xylopyranosyl oleanolate (**12b**)

OA (100 mg, 0.219 mmol), benzoylbromoglycoside **10b** (138 mg, 0.263 mmol), and Bu_4_NBr (4 mg) were dissolved in CHCl_3_ (4.0 mL); K_2_CO_3_ (152 mg) was dissolved in water (1.5 mL). The two solutions were mixed together and stirred vigorously at 50°C for 2.5 h. The organic layer was separated and diluted with CHCl_3_ (10 mL) and washed by water (10 mL × 2) and concentrated to give a brown residue, which was subjected to a column chromatography (2:1, petroleum ether-EtOAc) to afford oleanolic acid 2,3,4-tri-*O*-benzoyl-*β*-D-xylopyranosyl ester (**11b**) (183 mg, 92%) as a white foam. *R*_f_ = 0.32 (3:1, petroleum-EtOAc); ^1^H-NMR (CDCl_3_) δ 8.01-7.32 (*m*, 15H, H-C(Ar)), 5.89 (*m*, 2H), 5.63 (*dd*, 1H, *J=*8.8, 7.4), 5.40 (*td*, 1H, *J=*8.8, 5.2), 5.28 (*t*, 1H, *J=*3.3, H-C(12)), 4.42 (*dd*, 1H, *J=*11.9, 5.1), 3.74 (*dd*, 1H, *J=*11.9, 8.9), 3.16 (*dd*, 1H, *J=*11.1, 4.2, H-C(3)), 2.81 (*dd*, 1H, *J=*13.4, 3.8, H-C(18)), 0.97, 0.95, 0.87, 0.85, 0.84, 0.76, 0.49 (*s*, 7×3H, CH_3_); ESI-MS: 918.8 [(M+NH_4_)^+^].

Compound **11b** (190 mg, 0.211 mmol) was dissolved in dry CH_2_Cl_2_-MeOH (1:2, 6 mL) and a freshly prepared solution of NaOMe in MeOH (1.0 M, 0.50 mL) was added. The mixture was stirred at rt for 2 h and then neutralized with Dowex H^+^ resin to pH 7 and filtered. The filtrate was concentrated and purified with a silica gel column chromatography (12:1, CHCl_3_-MeOH) to give *β*-D-xylopyranosyl oleanolate (**12b**) (100 mg, 80%) as a white powder. *R*_f_ = 0.50 (10:1, CHCl_3_-MeOH); ^1^H-NMR (pyridine-*d_5_*) δ 6.24 (*d*, 1H, *J=*6.7, H-C(1')), 5.46 (*br s*, 1H, H-C(12)), 4.38 (*dd*, 1H, *J=*11.7, 4.4), 4.22 (*m*, 3H), 3.83 (*m*, 1H), 3.43 (*m*, 1H, H-C(3)), 3.25 (*dd*, 1H, *J=*11.8, 2.6, H-C(18)), 1.23 (*s*, 6H, 2×CH_3_), 1.10, 1.02, 0.92 (*s*, 3×3H, CH_3_), 0.90 (*s*, 6H, 2×CH_3_); ^13^C-NMR (pyridine-*d_5_*) δ 176.6, 144.1, 123.0, 96.3, 78.4, 78.1, 73.7, 70.9, 67.8, 55.8, 48.1, 47.2, 46.2, 42.2, 41.7, 39.9, 39.4, 38.9, 37.4, 34.0, 33.2, 33.1, 32.8, 30.8, 28.8, 28.3, 28.1, 26.1, 23.8, 23.6, 23.4, 18.8, 17.5, 16.6, 15.6; ESI-MS: 611.5 [(M+Na)^+^]. HRMS: *m*/*z* 587.3950 [M-H]^-^ ([C_35_H_55_O_7_] = 587.3953).

*β*-D-Galactopyranosyl oleanolate (**9a**), *α*-D-glucopyranosyl-(1→4)-*β*-D-glucopyranosyl oleanolate (**12c**) and *α*-L-rhamnopyranosyl oleanolate (**9b**) were also prepared according to the same procedure described for 12b. The analytical data of **9a** and **9b** were identical to that of **9a** and **9b** prepared by approach 1.

*α-D-Glucopyranosyl-(1→4)-β-D-glucopyranosyl oleanolate* (**12c**). *R*_f_ = 0.23 (4:1, CHCl_3_-MeOH); ^1^H-NMR (pyridine-*d_5_*) δ 6.23 (*d*, 1H, *J=*8.0, H-C(1')), 5.95 (*d*, 1H, *J=*3.2, H-C(1'')), 5.44 (*br s*, 1H, H-C(12)), 4.61-4.53 (*m*, 3H), 4.49 (*m*, 2H), 4.41-4.32 (*m*, 3H), 4.20-4.15 (*m*, 3H), 3.87 (*br d*, 1H, *J=*8.8), 3.43 (*m*, 1H, H-C(3)), 3.19 (*br d*, 1H, *J=*10.4, H-C(18)), 1.22 (*s*, 6H, 2×CH_3_), 1.10, 1.02 (*s*, 2×3H, CH_3_), 0.90 (*s*, 6H, 2×CH_3_), 0.88 (*s*, 3H, CH_3_); ^13^C-NMR (pyridine-*d_5_*) δ 176.4, 144.1, 123.2, 103.1, 95.5, 80.6, 78.3, 78.1, 77.6, 75.5, 75.4, 74.4, 73.6, 71.9, 62.7, 61.4, 55.8, 48.2, 47.0, 46.2, 42.2, 41.8, 39.9, 39.4, 39.0, 37.4, 34.0, 33.2, 33.2, 32.6, 30.8, 28.8, 28.3, 28.1, 26.1, 23.9, 23.7, 23.4, 18.8, 17.5, 16.6, 15.7; ESI-MS: 779.2 [(M-H)^-^]. HRMS: *m*/*z* 779.4585 [M-H]^-^ ([C_42_H_67_O_13_] = 779.4587).

### Synthesis of OA 3,28-diglycosides **14a~14b**, approach 1: β-D-glucopyranosyl oleanolate 3-O-α-L-arabinopyranoside (**14b**).

A mixture of intermediate **5d** (290 mg, 0.322 mmol), trichloroacetimidate **3a** (286 mg, 0.386 mmol) and powdered 4 Å molecular sieves (1 g) in dry CH_2_Cl_2_ (8 mL) were stirred for 30 min at 0°C. A solution of TMSOTf in dry CH_2_Cl_2_ (5%, 0.50 mL) was added dropwise and the mixture was warmed to rt and stirred for 45 min before quenching the reaction with Et_3_N (0.10 mL). The mixture was then filtered and the filtrate was concentrated and purified by a silica gel column chromatography (3:1, petroleum ether-EtOAc) to afford 2,3,4,6-tetra-*O*-benzoyl-*β*-D-glucopyranosyl oleanolate 3-*O*-(2,3,4-tri-*O*-benzoyl-*α*-L-arabinopyranoside) (**13b**) (438 mg, 92%) as a white powder. *R*_f_ = 0.22 (3:1, petroleum ether-EtOAc); ^1^H-NMR δ: (CDCl_3_) δ 8.08-7.25 (*m*, 35H, H-C(Ar)), 5.96 (*dd*, 1H, *J=*19.2, 9.7), 5.93 (*d*, 1H, *J=*8.3, H-C(1'')), 5.78-5.66 (*m*, 4H), 5.58 (*dd*, 1H, *J=*8.9, 3.4), 5.27 (*br s*, 1H, H-C(12)), 4.75 (*d*, 1H, *J=*6.5, H-C(1')), 4.56-4.43 (*m*, 2H), 4.34-4.23 (*m*, 2H), 3.86 (*br d*, 1H, *J=*11.6), 3.10 (*dd*, 1H, *J=*11.1, 4.8, H-C(3)), 2.77 (*br d*, 1H, *J=*10.9, H-C(18)), 0.93, 0.85, 0.82, 0.73, 0.73, 0.62, 0.41 (*s*, 7×3H, CH_3_); ESI-MS: 1501.9 [(M+Na)^+^].

Compound **13b** (396 mg, 0.268 mmol) was dissolved in dry CH_2_Cl_2_-MeOH (1:2, 30 mL), to which a freshly prepared solution of NaOMe in MeOH (1.0 M, 1.20 ml) was added. The mixture was stirred at rt for 3 h and then neutralized with Dowex H^+^ resin to pH 7. The resin was then removed by filtration. Recrystallization with MeOH-Et_2_O furnished *β*-D-glucopyranosyl oleanolate 3-*O*-*α*-L-arabinopyranoside (**14b**) (133 mg, 66%) as a white powder. *R*_f_ = 0.46 (4:1, CHCl_3_-MeOH); ^1^H-NMR (pyridine-*d_5_*) δ 6.33 (*d*, 1H, *J=*7.9, H-C(1'')), 5.42 (*br s*, 1H, H-C(12)), 4.75 (*d*, 1H, *J=*7.1, H-C(1')), 4.45-4.37 (*m*, 4H), 4.33-4.28 (*m*, 3H), 4.17-4.13 (*m*, 2H), 4.03 (*m*, 1H), 3.82 (*br d*, 1H, *J=*10.7), 3.34 (*m*, 1H, H-C(3)), 3.19 (*dd*, 1H, *J=*11.9, 2.0, H-C(18)), 1.26, 1.24, 1.09, 0.94, 0.90, 0.88, 0.86 (*s*, 7×3H, CH_3_); ^13^C-NMR (pyridine-*d_5_*) δ 176.4, 144.1, 122.9, 107.5, 95.8, 88.7, 79.3, 78.9, 74.6, 74.2, 72.9, 71.2, 69.5, 66.7, 62.3, 55.9, 48.1, 47.1, 46.3, 42.2, 41.8, 39.9, 39.6, 38.9, 37.1, 34.1, 33.2, 33.1, 32.6, 30.8, 28.3, 28.3, 26.6, 26.1, 23.9, 23.7, 23.5, 18.6, 17.5, 16.9, 15.6; ESI-MS: 785.2 [(M+Cl)^-^]. HRMS: *m*/*z* 750.4485 [M-H]^-^ ([C_41_H_65_O_12_] = 750.4481).

### β-D-Glucopyranosyl oleanolate 3-O-β-D-galactopyranoside (**14a**).

This substance was prepared according to the same procedure described for **14b**. *R*_f_ = 0.24 (4:1, CHCl_3_-MeOH); ^1^H-NMR (pyridine-*d_5_*) δ 6.36 (*d*, 1H, *J=*7.9, H-C(1'')), 5.45 (*br s*, 1H, H-C(12)), 4.88 (*d*, 1H, *J=*7.7, H-C(1')), 4.61 (*d*, 1H, *J=*3.2), 4.51-4.43 (*m*, 5H), 4.37-4.25 (*m*, 3H), 4.20-4.15 (*m*, 2H), 4.06 (*m*, 1H), 3.39 (*dd*, 1H, *J=*11.6, 3.6, H-C(3)), 3.22 (*dd*, 1H, *J=*13.2, 2.4, H-C(18)), 1.32, 1.29, 1.12, 0.98, 0.93, 0.90, 0.86 (*s*, 7×3H, CH_3_); ^13^C-NMR (pyridine-*d_5_*) δ 176.4, 144.2, 122.9, 107.5, 95.8, 88.8, 79.3, 78.9, 76.8, 75.5, 74.2, 73.2, 71.2, 70.3, 62.5, 62.3, 55.9, 48.1, 47.0, 46.3, 42.2, 41.8, 40.0, 39.6, 38.8, 37.0, 34.1, 33.2, 33.2, 32.6, 30.8, 28.3, 28.3, 26.7, 26.2, 23.8, 23.7, 23.5, 18.6, 17.5, 17.0, 15.6; ESI-MS: 781.0 [(M+H)^+^], 1561.1 [(2M+H)^+^]. HRMS: *m*/*z* 779.4580 [M-H]^-^ ([C_42_H_67_O_13_] = 779.4587).

### Synthesis of OA 3,28-diglycosides **16a~16b**, approach 2: β-D-xylopyranosyl oleanolate 3-O-β-D-galactopyranoside (**16a**).

A mixture of intermediate **11b** (600 mg, 0.666 mmol), trichloroacetimidate **3b** (570 mg, 0.765 mmol) and powdered 4 Å molecular sieves (600 mg) in dry CH_2_Cl_2_ (9 mL) were stirred for 30 min at rt. A solution of TMSOTf in dry CH_2_Cl_2_ (5%, 128 μL) was added dropwise and the mixture was stirred for 30 min before Et_3_N (0.40 ml) was added to quench the reaction. The mixture was then filtered and the filtrate was concentrated and purified by a silica gel column chromatography (3:1, petroleum ether-EtOAc) to afford 2,3,4-tri-*O*-benzoyl-*β*-D-xylopyranosyl oleanolate 3-*O*-(2,3,4,6-tetra-*O*-benzoyl-*β*-D-galactocopyranoside) (**15a**) (920 mg, 93.4%) as a white powder. *R*_f_ = 0.23 (3:1, petroleum ether-EtOAc); ^1^H-NMR (CDCl_3_) δ 8.12-7.22 (*m*, 35H, H-C(Ar)), 5.95 (*s*, 1H, H-C(4')), 5.90 (*t*, 1H, *J=*8.8, H-C(2'')), 5.87 (*d*, 1H, *J=*7.4, H-C(1'')), 5.83 (*t*, 1H, *J=*9.2, H-C(2')), 5.64-5.59 (*m*, 2H, H-C(3'), H-C(3'')), 5.40 (*m*, 1H, H-C(4'')), 5.30 (*br s*, 1H, H-C(12)), 4.81 (*d*, 1H, *J=*7.9, H-C(1')), 4.65 (*dd*, 1H, *J=*11.1, 7.7, H-C(6')-1), 4.44-4.41 (*m*, 2H, H-C(6')-2, H-C(5'')-1), 4.30 (*m*, 1H, H-C(5')), 3.72 (*dd*, 1H, *J=*14.7, 6.8, H-C(5'')-2), 3.09 (*dd*, 1H, *J=*11.7, 4.3, H-C(3)), 2.81 (*br d*, 1H, *J=*10.8, H-C(18)), 0.94, 0.88, 0.87, 0.79, 0.67, 0.66, 0.44 (*s*, 7×3H, CH_3_); ESI-MS: 1478.0 [(M-H)^-^], 1501.9 [(M+Na)^+^].

Compound **15a** (340 mg, 0.230 mmol) was dissolved in dry CH_2_Cl_2_-MeOH (1:2, 36 mL), to which a freshly prepared solution of NaOMe in MeOH (1.0 M, 1.60 mL) was added. The solution was stirred at rt for 12 h and then neutralized with Dowex H^+^ resin to pH 7. The resin was then removed by filtration. Recrystallization with MeOH-Et_2_O furnished *β*-D-xylopyranosyl oleanolate 3-*O*-*β*-D-galactopyranoside (**16a**) (132 mg, 76.5%) as a white powder. *R*_f_ = 0.41 (4:1, CHCl_3_-MeOH); ^1^H-NMR (pyridine-*d_5_*) δ 6.22 (*d*, 1H, *J=*6.4, H-C(1'')), 5.43 (*br s*, 1H, H-C(12)), 4.86 (*d*, 1H, *J=*7.6, H-C(1')), 4.58 (*s*, 1H, H-C(4')), 4.48-4.42 (*m*, 3H, H-C(2'), H-C(6')-1, H-C(3'')), 4.36 (*dd*, 1H, *J=*11.6, 4.3, H-C(5'')-1), 4.22 (*m*, 1H, H-C(4'')), 4.21-4.15 (*m*, 3H, H-C(3'), H-C(6')-2, H-C(2'')), 4.12 (*m*, 1H, H-C(5')), 3.80 (*m*, 1H, H-C(5'')-2), 3.36 (*dd*, 1H, *J=*18.7, 6.0, H-C(3)), 3.23 (*br d*, 1H, *J=*10.4, H-C(18)), 1.29, 1.25, 1.06, 0.96, 0.92, 0.90, 0.83 (*s*, 7×3H, CH_3_); ^13^C-NMR (pyridine-*d_5_*) δ 176.6, 144.1, 122.9, 107.6, 96.3, 88.7, 78.4, 76.9, 75.5, 73.7, 73.2, 70.9, 70.3, 67.8, 62.5, 55.8, 48.0, 47.1, 46.2, 42.1, 41.7, 39.9, 39.5, 38.7, 36.9, 34.0, 33.1, 33.1, 32.7, 30.8, 28.2, 28.2, 26.7, 26.1, 23.8, 23.6, 23.3, 18.5, 17.5, 17.0, 15.5; ESI-MS: 773.4 [(M+Na)^+^]. HRMS: *m*/*z* 750.4483 [M-H]^-^ ([C_41_H_65_O_12_] = 750.4481).

### α-D-Glucopyranosyl-(1→4)-β-D-glucopyranosyl oleanolate 3-O-β-D-xylopyranoside (**16b**).

This compound was prepared according to the same procedure described for **16a**. *R*_f_ = 0.19 (4:1, CHCl_3_-MeOH); ^1^H-NMR (pyridine-*d_5_*) δ 6.22 (*d*, 1H, *J=*8.2, H-C(1'')), 5.94 (*d*, 1H, *J=*3.5, H-C(1''')), 5.42 (*br s*, 1H, H-C(12)), 4.82 (*d*, 1H, *J=*7.5, H-C(1')), 4.58-4.53 (*m*, 3H), 4.45 (*m*, 2H), 4.37 (*m*, 3H), 4.33 (*t*, 1H, *J=*5.8), 4.22 (*m*, 1H), 4.17-4.14 (*m*, 4H), 4.01 (*t*, 1H, *J=*8.2), 3.87 (*br d*, 1H, *J=*8.2), 3.77 (*t*, 1H, *J=*11.5), 3.34 (*dd*, 1H, *J=*11.7, 4.2, H-C(3)), 3.17 (*dd*, 1H, *J=*10.9, 3.9, H-C(18)), 1.29, 1.25, 1.07, 0.98, 0.90, 0.88, 0.86 (*s*, 7×3H, CH_3_); ^13^C-NMR (pyridine-*d_5_*) δ 176.4, 144.1, 123.0, 107.8, 103.1, 95.5, 88.7, 80.6, 78.7, 78.3, 77.6, 75.6, 75.4, 75.4, 74.5, 73.6, 71.9, 71.3, 67.2, 62.8, 61.4, 55.9, 48.1, 47.0, 46.2, 42.2, 41.8, 40.0, 39.6, 38.8, 37.1, 34.0, 33.2, 33.2, 32.6, 30.8, 28.2, 28.2, 26.8, 26.1, 23.9, 23.7, 23.4, 18.5, 17.5, 17.0, 15.6; ESI-MS: 935.5 [(M+Na)^+^]. HRMS: *m*/*z* 911.5011 [M-H]^-^ ([C_47_H_75_O_17_] = 911.5011).

## References

[1] Dzubak P., Hajduch M., Vydra D., Hustova A., Kvasnica M., Biedermann D., Markova L., Urban M., Sarek J. (2006). Pharmacological activities of natural triterpenoids and their therapeutic implications. Nat. Prod. Rep..

[2] Liu J. (1995). Pharmacology of oleanolic acid and ursolic acid. J. Ethnopharmacol..

[3] Hsu H.Y., Yang J.J., Lin C.C. (1997). Effects of oleanolic acid and ursolic acid on inhibiting tumor growth and enhancing the recovery of hematopoietic system postirradiation in mice. Cancer Lett..

[4] Barthomeuf C., Debiton E., Mshvidadze V., Kemertelidze E., Balansard G. (2002). In vitro activity of hederacolchisid A1 compared with other saponins from Hedera colchica against proliferation of human carcinoma and melanoma cells. Planta Med..

[5] Jung H.J., Lee C.O., Lee K.T., Choi J., Park H.J. (2004). Structure-activity relationship of oleanane disaccharides isolated from Akebia quinata versus cytotoxicity against cancer cells and NO inhibition. Biol. Pharm. Bull..

[6] Li Y., Matsuda H., Yoshikawa M. (1999). Effects of oleanolic acid glycosides on gastrointestinal transit and ileus in mice. Bioorg. Med. Chem..

[7] Hardegger E., Leemann H.J., Robinet F.G. (1952). Steroid and triterpene glycosides. III. Glucosides of esters of oleanic acid and glycosidal binding in beet-sugar saponins. Helv. Chim. Acta..

[8] Kochetkov N.K., Khorlin A.Ya., Snyatkova V.I. (1964). Triterpenoid saponins 13. Halolysis of glycosides of the triterpene series and the synthesis of glucosides of oleanolic acid. Izvestiya Akademii Nauk SSSR, Seriya Khimicheskaya..

[9] Kochetkov N.K., Khorlin A.Ya., Bochkov A.F., Kretsu L.G. (1966). Synthesis of glycosides of oleanolic acid. Izvestiya Akademii Nauk SSSR, Seriya Khimicheskaya..

[10] Juodvirsis A., Troshchenko A.T. (1966). Synthesis of triterpene glycosides I. Synthesis of acyl glycosides of oleanolic and ursotic acids. Khimiya Prirodnykh Soedinenii..

[11] Janiszowska W., Wilkomirski B., Kasprzyk Z. (1980). Synthesis of oleanolic acid 3-O-monoglucoside. Polish Journal of Chemistry..

[12] Seebacher W., Haslinger E., Rauchensteiner K., Jurenitsch J., Presser A., Weis R. (1999). Synthesis and Haemolytic Activity of Randianin Isomers. Monatshefte fuer Chemie..

[13] Seebacher W., Weis R., Jurenitsch J., Rauchensteiner K., Haslinger E. (2000). Synthesis and hemolytic properties of arvensoside B isomers. Monatshefte fuer Chemie..

[14] Ohtani K., Mizutani K., Kasai R., Tanaka O. (1984). Selective cleavage of ester type glycoside-linkages and its application to structure determination of natural oligoglycosides. Tetrahedron Lett..

[15] Deng S.J., Yu B., Xie J.M., Hui Y.Z. (1999). A highly efficient glycosylation of sapogenins. J. Org. Chem..

[16] Schmidt R.R., Michel J. (1980). Facile synthesis of α- and β-O-glycosyl imidates;Preparation of glycosides and disaccharides. Angew. Chem. Int. Ed. Engl..

[17] Zang J., Li Y.X., Li C.X., Song N., Yue C.L. (2005). Synthesis of Four Oleanolic Saponins. Periodical of Ocean University of China..

[18] Sun J., Han X., Yu B. (2003). Synthesis of a typical *N*-acetylglucosamine containing saponin, oleanolic acid 3-*O*-α-L-arabinopyranosyl-(1→2)-α-L-arabinopyranosyl-(1→6)-2-acetamido-2-deoxy-β-D-glucopyranoside. Carbohydr. Res..

[19] Cheng M.S., Yan M.C., Liu Y., Zheng L.G., Liu J. (2006). Synthesis of beta-hederin and Hederacolchiside A. 1: triterpenoid saponins bearing a unique cytotoxicity-inducing disaccharide moiety. Carbohydr. Res..

[20] Li C.X., Zang J., Wang P., Zhang X.L., Guan H.S., Li Y.X. (2006). Synthesis of two natural oleanolic acid saponins. Chinese J. Chem..

[21] Yu B., Xie J., Deng S., Hui Y. (1999). First Synthesis of a bidesmosidic triterpene saponin by a highly efficient procedure. J. Am. Chem. Soc..

[22] Peng W., Sun J., Lin F., Han X., Yu B. (2004). Facile Synthesis of Ginsenoside Ro. Synlett.

[23] Yan M.C., Liu Y., Chen H., Ke Y., Xu Q.C., Cheng M.S. (2006). Synthesis and antitumor activity of two natural *N*-acetylglucosamine-bearing triterpenoid saponins: Lotoidoside D and E. Bioorg. Med. Chem. Lett..

[24] Winterstein A., Stein G. (1931). Double bond between C-12 and C-13 of oleanolic acid is inert to catalytic hydrogenation. Z. Physiol. Chem..

[25] Bliard C., Massiot G., Nazabadioko S. (1994). Glycosylation of acids under phase transfer conditions. Partial synthesis of saponins. Tetrahedron Lett..

[26] Schmidt R.R., Kinzy W. (1994). Anomeric-oxygen activation for glycoside synthesis: The trichloroacetimidate method. Adv. Carbohydr. Chem. Biochem..

